# A text-mining approach to study the real-world effectiveness and potentially fatal immune-related adverse events of PD-1 and PD-L1 inhibitors in older patients with stage III/IV non-small cell lung cancer

**DOI:** 10.1186/s12885-023-10701-z

**Published:** 2023-03-14

**Authors:** Hanieh Abedian Kalkhoran, Juliëtte Zwaveling, Bert N. Storm, Sylvia A. van Laar, Johanneke EA Portielje, Henk Codrington, Dieuwke Luijten, Pepijn Brocken, Egbert F. Smit, Loes E. Visser

**Affiliations:** 1 Department of Clinical Pharmacy and Toxicology, Leiden University Medical Centre, Leiden, The Netherlands; 2 Department of Pharmacy, Haga Teaching Hospital, The Hague, The Netherlands; 3 Department of Internal Medicine – Medical Oncology, Leiden University Medical Centre, Leiden, The Netherlands; 4 Department of Pulmonary Diseases – Pulmonic Oncology, Haga Teaching Hospital, The Hague, The Netherlands; 5 Department of Pulmonary Disease, Leiden University Medical Centre, Leiden, The Netherlands; 6 Department of Hospital Pharmacy, Erasmus Medical Centre, Rotterdam, The Netherlands; 7 Department of Epidemiology, Erasmus Medical Centre, Rotterdam, The Netherlands

**Keywords:** Non-small cell lung cancer (NSCLC), Immune checkpoint inhibitor (ICI), Anti-PD-(L)1 therapy, Real-world, Elderly

## Abstract

**Background:**

This study was designed to investigate the impact of age on the effectiveness and immune-related adverse events (irAEs) of programmed death-(ligand)1 [PD-(L)1] inhibitors in patients with non-small cell lung cancer (NSCLC) using a novel text-mining technique.

**Methods:**

This retrospective study included patients with stage III/IV NSCLC treated with a PD-(L)1 inhibitor (nivolumab, pembrolizumab, atezolizumab and durvalumab) at Leiden University Medical Centre and Haga Teaching hospital, (both in The Netherlands) from September 2016 to May 2021. All the relevant data was extracted from the structured and unstructured fields of the Electronic Health Records using a novel text-mining tool. Effectiveness [progression-free survival (PFS) and overall survival (OS)] and safety (the incidence of nine potentially fatal irAEs and systemic corticosteroid requirement) outcomes were compared across age subgroups (young: < 65 years, Middle-aged: 65–74 years, and old: ≥ 75 years) after adjustment for confounding.

**Results:**

Of 689 patients, 310 patients (45.0%) were < 65 years, 275 patients (39.9%) were aged between 65 and 74 years, and 104 patients (15.1%) were ≥ 75 years. There was no significant difference between younger and older patients regarding PFS (median PFS 12, 8, 13 months respectively; Hazard ratio (HR)_middle-aged_ = 1.14, 95% CI 0.92–1.41; HR_old_ = 1.10, 95% CI 0.78–1.42). This was also the case for OS (median OS 19, 14, 18 months respectively; HR_middle-aged_ = 1.22, 95% CI 0.96–1.53; HR_old_ = 1.10, 95% CI 0.79–1.52). Safety analysis demonstrated a higher incidence of pneumonitis among patients aged 65–74. When all the investigated irAEs were pooled, there was no statistically significant difference found between age and the incidence of potentially fatal irAEs.

**Conclusions:**

The use of PD-(L)1 inhibitors is not associated with age related decrease of PFS and OS, nor with increased incidence of serious irAEs compared to younger patients receiving these treatments. Chronological age must therefore not be used as a predictor for the effectiveness or safety of ICIs.

**Supplementary Information:**

The online version contains supplementary material available at 10.1186/s12885-023-10701-z.

## Background

Lung cancer is the leading cause of cancer-related mortality worldwide, with 2.21 million new cases and approximately 1.8 million deaths reported globally in 2020 [1]. The poor prognosis of this disease is largely due to its often late diagnosis at advanced or metastatic stage. Incidence increases significantly with age, with a median age of 70 years old at diagnosis [2]. Non–small cell lung cancer (NSCLC) accounts for approximately 85% of all lung cancer cases [3].

The introduction of immune checkpoint inhibitors (ICIs), in particular anti-programmed cell death-protein 1 (anti-PD-1, nivolumab and pembrolizumab) and anti-programmed death ligand-1 (anti-PD-L1, atezolizumab and durvalumab) antibodies, has revolutionised the management of NSCLC in the last decade. PD-(L)1 inhibitors, as monotherapy or in combination with chemotherapy, are currently indicated for the first-line treatment or as consolidation therapy of stage III and IV NSCLC.

Several randomised clinical trials (RCTs) and observational studies have shown improved patient outcomes in terms of progression-free survival (PFS) and overall survival (OS) compared to conventional cancer treatment options [4–8]. Additionally, the safety profile of PD-(L)1 inhibitors appears to be more favourable compared to cytotoxic chemotherapy agents [9]. However, due to the activation of autoreactive T cells in a variety of host tissues, ICIs are associated with a considerable risk of immune-related adverse events (irAEs). These irAEs can affect any organ, but are mainly detected in colon, liver, lungs, pituitary, thyroid and skin [10]. Additionally, several reports have been made about uncommon, potentially fatal irAEs during the past years [11–14]. In fact, ICIs have been associated with potentially fatal toxicities in 0.4% to 1.2% of the patients [15]. Due to the scarcity of these fatal irAEs, the exact estimation of their incidence is challenging. According to an analysis from the World Health Organization (WHO) pharmacovigilance database, colitis, pneumonitis, hepatitis, myocarditis, myositis, nephritis, myasthenia gravis, encephalitis and meningitis are among the most common fatal toxic effects associated with PD-(L)1 inhibitors [16]. All the aforementioned adverse events are listed as potential irADRs in the summary of product characteristics (SmPC) of PD-(L)1 inhibitors.

While older adults constitute the majority of NSCLC patients in clinical practice, our knowledge on effectiveness and safety of many novel treatment options in elderly patients has remained limited due to their underrepresentation in clinical trials. Despite the fact that over 60% of patients with NSCLC receiving ICIs in clinical practice are older than 65 years, this group only made up 8% of the clinical trial participants in the CheckMate 017 and 057 trials [4, 5] and 10–15% in the Keynote 042 and 024 trials [16, 17]. It is worth mentioning that the results of a small subgroup analysis (*n* = 29) in CheckMate 057 [5] were suggestive of a decreased efficacy of nivolumab in patients aged 75 years or older (HR: 1.76;95% CI = 0.77–4.05). Moreover, older adults who are enrolled in clinical trials are typically fitter than the general geriatric patient population in clinical practice. In addition, aging has been associated with structural and functional deterioration of the immune system, known as ‘immunosenescence’. Immunosenescence could alter the effectiveness and degree of toxicities of immunotherapy in elderly patients [18]. Although, traditionally, older people are defined as those aged 65 years or over, within pharmacoepidemiologic studies, there is no formal cut-off age determined to define the “older patients”. Previous effectiveness and safety studies of “elderly” or “geriatric” patients receiving immunotherapy have used age cut-offs ranging from 60 to 80 years [8, 19, 20]. This variation may reflect the fact that immunosenescence is a gradual process. Clearly, the arbitrary definitions of chronological age for studying older patients do not show a true picture of the reality. However, certain cut-off points have to be defined to be able to conduct a comparative study.

The disparity between the trial participants and patients receiving ICIs in clinical practice reflects a potential knowledge gap which can largely be bridged by utilizing real-world evidence (RWE). Electronic health record (EHR) platforms are rich sources of RWE as ample information on therapies, diagnoses, laboratory test results, physicians’ notes, et cetera are easily and reliably accessible. One key challenge with the EHR data is the utilization of the free-text notes.

Recently, the introduction of Natural Language Processing (NLP) and text mining techniques has eased the extraction of free-text data. The rule-based text-mining software (IQVIA Patient Finder Solution-CTcue B.V., Amsterdam, the Netherlands) is an NLP-based tool, which can be used to extract structured as well as unstructured information from the EHRs on, among others, comorbidities, cancer-related variables, clinical outcomes and adverse events. A validation study using the CTcue showed an accuracy of 88.1–100% for the retrieval of main treatment outcomes such as PFS and OS from the EHRs when compared to manual reviewing process [21].

The current study aimed to investigate the effect of chronological age on the real-world effectiveness and immune-related safety of PD-(L)1 inhibitors in stage III and IV NSCLC using text-mining techniques.

## Methods

### Study design

This retrospective cohort study evaluated patients with stage III/IV NSCLC treated with any anti-PD-(L)1 antibodies in two Dutch hospitals (Leiden University Medical Center [LUMC], Leiden, and Haga Teaching hospital, The Hague) between September 1, 2016, and May 1, 2021.

The local Medical Ethics Review Committees (METC Leiden Den Haag Delft) of both hospitals reviewed this research and granted a waiver of informed consent. Additionally a non-WMO acknowledgement and a no objection certificate were issued by the boards of directors.

### Study population

Patients, 18 years and older, with a Diagnosis Treatment Combination (DTC) code^1^ for NSCLC and at least one prescription for any one of the studied drugs between September 2016 and May 2021 were included in this study. Patients who opted out of the reuse of their clinical information for research purposes were excluded.

The cohort entry date (= index date) was defined as the first medication order for any one of the following drugs: nivolumab, pembrolizumab, atezolizumab or durvalumab.

### Data source and retrieval

The data were primarily obtained from the Electronic Health Record (EHR). Patient selection and data collection were performed using CTcue (IQVIA Patient Finder Solution-CTcue B.V., Amsterdam, The Netherlands) text-mining software. This text-mining tool is linked to the EHR and allows for a more efficient extraction of structured (e.g., medication prescriptions, laboratory results, vital status and basic demographic factors) and unstructured (e.g., radiology and pathology reports, medical letters and notes) data. The exact architecture of CTcue is discussed by Van Laar et al., 2020 [21].

All data regarding cytostatic treatments (including the line of therapy, first and last prescriptions, etc.) in the Haga hospital were manually retrieved from the Cytostatic Management System (CMS).

### Outcomes

The primary outcome of the study was PFS between different age groups; that is the time (in full months) between the index date and the first disease progression or death of any cause, whichever occurred first. The secondary outcome of effectiveness was overall survival (OS), defined as the time (in full months) from the index date until death from any cause. The recorded vital status as well as the date of decease (where applicable) of the patients were verified during the study. For patients who did not reach the abovementioned outcomes during follow-up, PFS and OS were censored at the date of last recorded medical note or the end of the study period (March 31, 2021 at Haga Teaching Hospital and May 31, 2021 at LUMC), whichever came first.

The safety outcomes of the study were (i) the first incidence of colitis, pneumonitis, hepatitis, myocarditis, myositis, nephritis, myasthenia gravis, encephalitis or meningitis (i.e. potentially fatal irAEs), as diagnosed by the treating physician in medical records; and (ii) the first systemic corticosteroid prescription for the treatment of an irAE during the period of immunotherapy until six months after discontinuation of treatment. Since the data collection was performed using a text-mining tool, we hypothesized that clinically relevant irAEs are treated with systemic corticosteroids. Patients receiving a systemic corticosteroid for other indications (e.g. COPD exacerbation) were identified and excluded from this analysis.

### Analysis

Effectiveness and safety were analysed in all patients who received at least one dose of any one of the PD-(L)1 inhibitors, with separate analyses for patients aged < 65, 65–74 and ≥ 75 years and for the overall patient population. The youngest cohort (i.e. < 65 years old) served as the comparator in all the analyses.

The collected data on demographic, disease- and therapy-related characteristics were summarised as median (range) for continuous variables and numbers (percentage) for categorical variables. The treatment-related data were tabulated for each drug treatment.

The Kaplan–Meier method was used to estimate PFS and OS; age subgroups were compared using the log-rank test. The associations between age and the effectiveness outcomes were also examined using univariable and multivariable Cox proportional hazards regression model to calculate HRs with 95% confidence interval (CI). The confounders for the multivariable Cox model were selected using the change-in-estimate method. Potential confounders that changed the univariate HR by 10% were included in the multivariable Cox model. The proportional hazard assumptions was tested using Schoenfeld residuals. Multiple imputation was performed for covariates with more than 15–35% missing data. When < 15% of values was missing, the subjects with the missing values were omitted. Subgroup analysis was performed with treatment line (1 vs. > 2) and PD-L1 expression (< 1% vs. > 1%).

The robustness of the effectiveness findings were controlled by analysing age as a continuous variable in order to determine the probability of PFS or survival per year of increased age.

For the analysis of the safety outcomes, differences between the age groups for the cumulative incidence of the individual potentially fatal irAEs were assessed using a χ^2^ test (reference group < 65 yrs).

The incidence rates and relative risks (RR) for the pooled irAEs and corticosteroid prescription during immunotherapy were calculated and reported as IR per 100 person years and RR ± 95% CI. The total person‐time at risk was determined by calculating the time interval between the index date and the occurrence of any one of the safety events, death from any cause, six months after discontinuation of treatment or the date of the latest health information or end of the data collection period, whichever occurred first.

The process of data retrieval from free-text note was validated by comparing the classification and timing of the results of the text-mining software with manual review in 10% of the total cohort. Per extracted cofactor, precision, recall and F_1_ scores were calculated [22]. Due to the lack of a formal cut-off point for an appropriate F_1_ score, a score of ≥ 80% was arbitrarily considered acceptable for the extracted covariates. Covariates with a lower F_1_ score were excluded from the analysis. Regarding the outcomes, lower F_1_ values, resulting from a low precision and a high recall, were also considered acceptable as the false positive findings could be manually removed after the initial generation of results in CTcue.

For all the statistical tests, a two-sided P-value of < 0.05 was considered statistically significant. All statistical analyses were performed using SPSS, version 24.

## Results

### Patient and treatment baseline characteristics

**Fig. 1:**
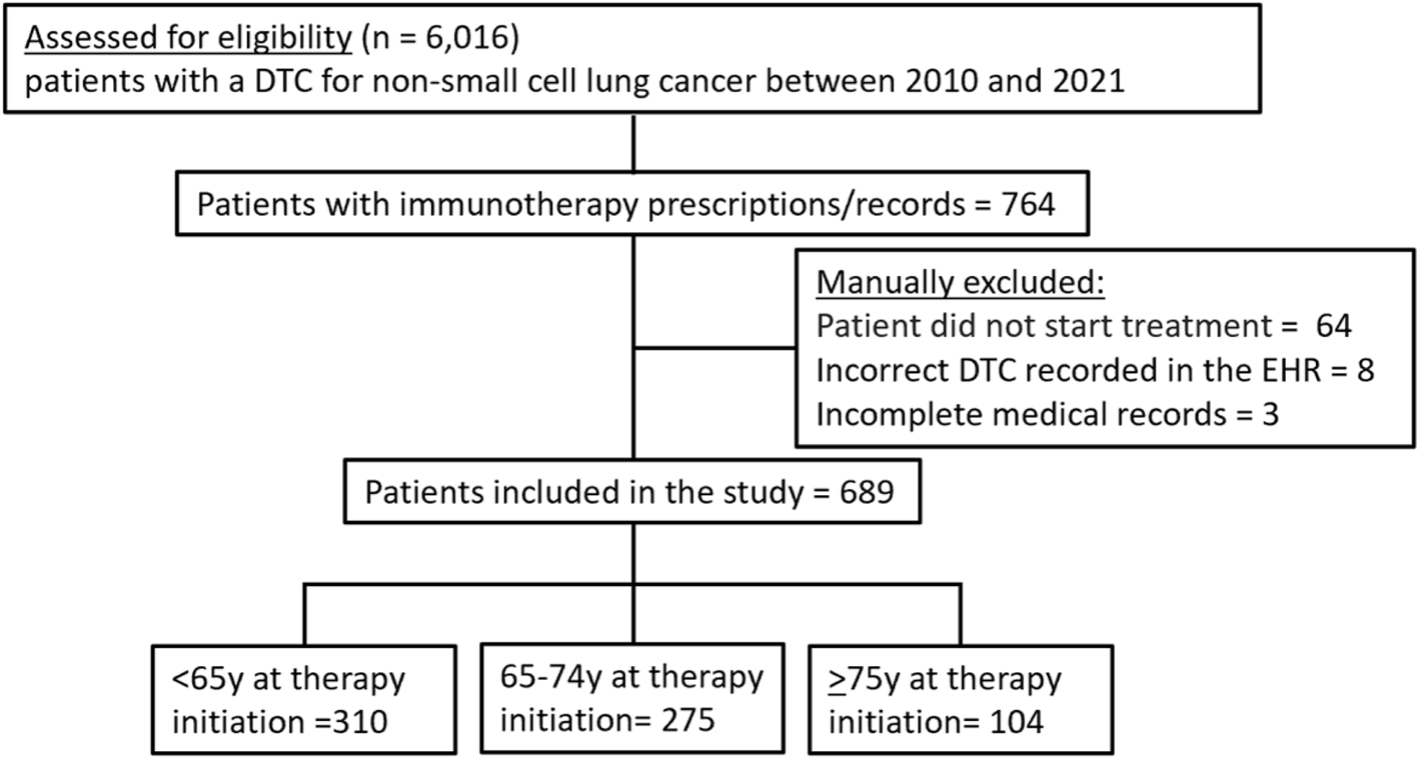
Study selection process

In total 689 patients with NSCLC and at least one prescription of a PD-(L)1 inhibitor were identified (Fig. 1). All patient-, disease- and therapy-related characteristics are summarised in Table 1.

**Table 1:** Patient-, disease- and therapy-related characteristics at index date ^a^

Characteristic	All patients (*N* = 689)	< 65 years (*N* = 310)	65–74 years (*N* = 275)	> 75 years (*N* = 104)
**Baseline patient characteristics**				
Sex, N (%)				
Female	308 (44.7)	156 (50.2)	115 (41.8)	37 (35.7)
Age, years, median (IQR)	66 (12.0)	59 (8.0)	69 (4.0)	78 (5.0)
BMI, kg/m^2^, median (IQR)	24.5 (6.0)	24.4 (6.0)	24.4 (6.3)	24.9 (5.3)
Missing = 236 (34.2%)				
Smoking status, N (%)				
Never-smoker	56 (8.1)	25 (8.1)	17 (6.2)	14 (13.5)
Former smoker ^b^	407 (59.1)	157 (50.6)	181 (65.8)	69 (66.3)
Current smoker	180 (26.1)	106 (34.2)	61 (22.2)	13 (12.5)
Missing	46 (6.7)	22 (7.1)	16 (5.8)	8 (7.7)
**Disease-related characteristics**				
Tumour histology, N (%)				
Adenocarcinoma	506 (73.4)	234 (75.5)	191 (69.5)	81 (77.9)
Squamous cell carcinoma	134 (19.5)	55 (17.7)	61 (22.2)	18 (18.3)
Large cell carcinoma	28 (4.1)	12 (3.9)	15 (5.5)	1 (1.0)
Missing	21 (3.0)	9 (2.9)	8 (2.9)	4 (3.8)
NSCLC stage, N (%)				
III	91 (13.2)	40 (12.9)	36 (13.1)	15 (14.4)
IV	528 (76.6)	238 (76.8)	207 (75.3)	83 (79.8)
Missing	70 (10.2)	32 (10.3)	32 (11.6)	6 (5.8)
CNS metastasis, N (%)	82 (11.9)	47 (15.2)	27 (9.8)	8 (7.7)
PD-L1 expression, N (%)				
Negative (< 1%)	254 (36.5)	121 (39.0)	112 (40.7)	21 (20.2)
Positive (1–49%)	178 (25.6)	78 (25.2)	70 (25.5)	30 (28.8)
Strongly positive (> 50%)	185 (26.9)	75 (24.2)	65 (23.6)	45 (43.3)
Missing	72 (10.4)	36 (11.6)	28 (10.2)	8 (7.7)
ECOG PS, N (%)				
0	236 (34.3)	122 (39.4)	85 (30.9)	29 (27.9)
1	184 (26.7)	73 (23.5)	76 (27.6)	35 (33.7)
2	79 (11.4)	29 (9.4)	30 (10.9)	20 (19.3)
Missing	190 (27.6)	86 (27.7)	84 (30.5)	20 (19.2)
Comorbidities, N (%)				
COPD	287 (41.7)	130 (41.9)	119 (43.3)	38 (36.5)
**Therapy-related characteristics**				
Treatment center, N (%)				
Haga Teaching Hospital	288 (41.8)	129 (41.6)	122 (44.4)	37 (35.6)
LUMC	401 (58.2)	181 (58.4)	153 (55.6)	67 (64.4)
Previous lobectomy, N (%)	56 (8.1)	31 (10.0)	20 (7.3)	5 (4.8)
Previous radiotherapy, N (%)	332 (48.2)	164 (52.9)	129 (46.9)	39 (37.5)
Immuno-radiotherapy, N (%)	260 (37.7)	129 (41.6)	97 (35.3)	34 (32.7)
Line of therapy, N (%)				
1	452 (65.6)	182 (58.7)	184 (66.9)	86 (82.7)
2	179 (25.9)	102 (32.9)	62 (22.5)	15 (14.4)
≥3	58 (8.4)	26 (8.3)	29 (10.5)	3 (2.9)
Immune checkpoint inhibitor, N (%)				
Pembrolizumab	420 (61.0)	172 (55.4)	170 (61.8)	78 (75.0)
Nivolumab	118 (17.1)	57 (18.4)	46 (16.7)	15 (14.4)
Atezolizumab	71 (10.3)	41 (13.2)	27 (9.8)	3 (2.9)
Durvalumab	80 (11.6)	40 (12.9)	32 (11.6)	8 (7.7)
Chemo-immunotherapy c, N (%)	251 (36.4)	115 (37.1)	104 (37.8)	32 (30.8)
Duration of therapy, N (%)				
Single treatment	57 (8.2)	22 (7.1)	23 (8.4)	12 (11.5)
2–90 days	241 (34.9)	104 (33.5)	107 (38.9)	30 (28.9)
91–180 days	124 (17.9)	52 (16.8)	53 (19.2)	19 (18.3)
181–365 days	114 (16.5)	54 (17.4)	42 (15.3)	18 (17.3)
> 365 days	153 (22.2)	78 (25.2)	50 (18.2)	25 (24.0)

^a^ First prescription of immunotherapy

^b^ Past user/smoker: Quitted > 3 months before start of immunotherapy

^c^ pembrolizumab in combination with carboplatin-pemetrexed, carboplatin-paclitaxel, cisplatin-pemetrexed, carboplatin-paclitaxel-bevacizumab, pemetrexed │ nivolumab in combination with ipilimumab │ atezolizumab in combination with carboplatin-paclitaxel-bevacizumab

The median age at the start of immunotherapy was 66 years (range 31–89). 310 patients (45.0%) were younger than 65 years, 275 patients (39.9%) were aged between 65 and 74 years, and 104 patients (15.1%) were 75 years and over. Most patients were male (55.3%), current or former-smokers (85.2%), with PS 0–1 (61.0%) and adenocarcinoma histology (73.4%).

The oldest group had the highest proportion of never-smokers (8.1% vs. 6.2% vs. 13.5%), a strongly positive PD-L1 expression (24.2%, 23.6% and 43.3%) and a poorer functional status (i.e. ECOG 2) at the start of immunotherapy (9.4% vs. 10.9% vs. 19.3%).

Over half of the patients received immunotherapy as first line treatment (65.6%) and this was most often the case among the oldest group of patients (58.7% vs. 66.9% vs. 82.7%). Of all treated patients, 251 (36.4%) received chemo-immunotherapy, which was approximately evenly distributed between the three age groups. Most patients in our cohort received pembrolizumab (61.0%), followed by nivolumab (17.1%), durvalumab (11.6%) and lastly atezolizumab (10.3%).

The duration of immunotherapy treatment was more or less equally divided between the three age groups, with 57 (8.2%) patients receiving only a single dose of treatment, 365 (52.8%) patients receiving treatment for at least 6 months, and 153 (22.2%) patients treated for longer than one year.

### Effectiveness outcomes

**Fig. 2:**
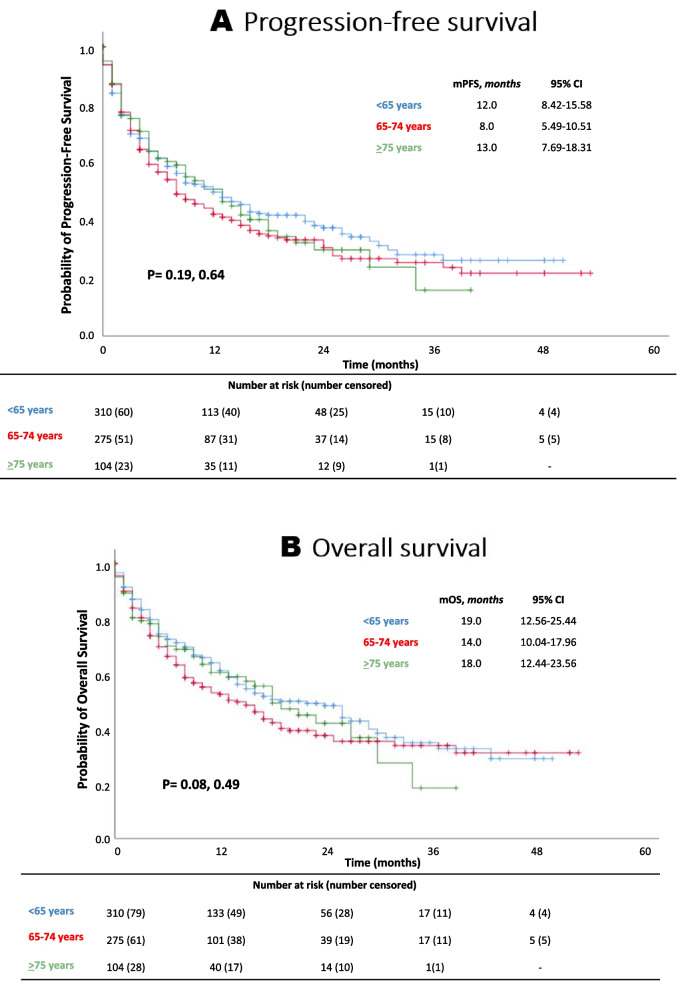
Kaplan–Meier survival plots according to age groups. (**A**) Progression free-survival, (**B**) overall survival. CI, confidence interval

The median PFS for patients under the age of 65 years, between 65 and 74 years and 75 years or older was 12 months (95% CI 8.42–15.58), 8 months (95% CI 5.49–10.51) and 13 months (95% CI 7.96–18.31), respectively (P_middle-aged_ = 0.19 and P_old_ = 0.64) (Fig. 2A). The median OS for these three age groups was 19 months (95% CI 12.56–25.44), 14 months (95% CI 10.04–17.96), and 18 months (95% CI 12.44–23.56), respectively (P_middle-aged_ = 0.08 and P_old_ = 0.49) (Fig. 2B).

**Fig. 3:**
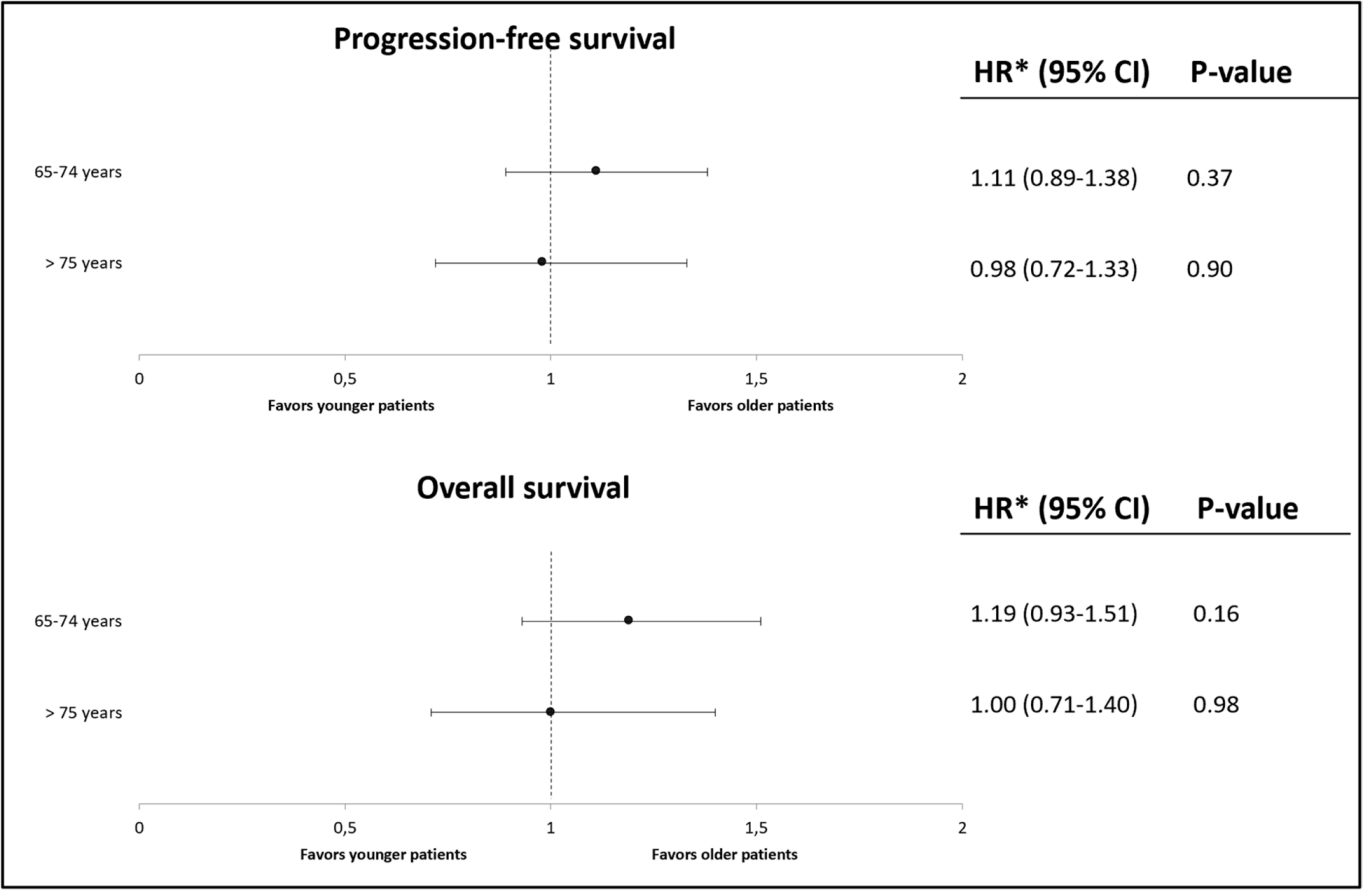
Multivariable Cox regression analysis: PFS and OS of patients aged 65–74 and > 75 years compared with younger patients (< 65 years). CI = confidence interval *HR adjusted for BMI, smoking status and ECOG PS

Similarly, there was no statistically significant difference in HRs for progression between the older and the younger patients (HR_middle-aged_ = 1.14, 95% CI 0.92–1.41; HR_old_ = 1.10, 0.78–1.42). This was also the case for OS (HR_middle-aged_ = 1.22, 95% CI 0.96–1.53; HR_old_ = 1.10, 0.79–1.52) (supplementary Table 1). The absence of a statistically significant association between age and the effectiveness of the PD-(L)1 inhibitors (i.e. PFS and OS) was confirmed after adjusting for ECOG PS, smoking status and BMI in the multivariate Cox proportional hazards regression model (Fig. 3).

The association between age and the effectiveness outcomes remained not statistically significant in subgroup analysis stratified by PD-L1 expression levels and line of treatment (supplementary Table 2). Age was also analysed as a linear variable; hazard ratios were 1.00 (0.99–1.02) and 1.01 (0.99–1.02) per year of increased age for PFS and OS respectively (supplementary Table 3).

### Safety outcomes

**Fig. 4:**
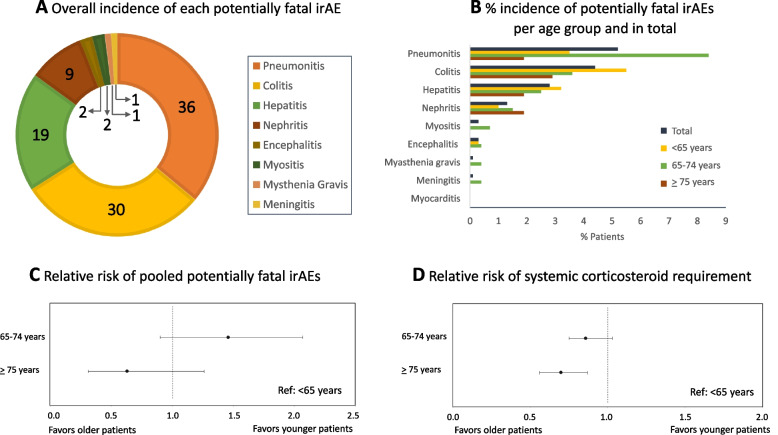
The safety outcomes between different age groups **A** the overall incidence of each of the nine studied potentially fatal irAEs (nr. of patients), **B** the percentage of patients experiencing one of the studied irAE per age subgroup and in total (%), **C** relative risk of pooled potentially fatal irAEs (reference: patients aged < 65 years), **D** relative risk of systemic corticosteroid therapy requirement during (+ 6 months after) immunotherapy (reference: patients aged < 65 years

In total 100 patients (14.5%) experienced one of the studied irAEs, with pneumonitis (5.2%), colitis (4.4%) and hepatitis (2.8%) being the most commonly occurring irAEs (Figs. 4A, B, supplementary Table 4). No cases of myocarditis or irAE-related deaths were recorded. Chi-squared analysis only showed a statistically higher incidence of pneumonitis among middle-aged patients compared to younger patients (*P* = 0.01). The incidence of all other irAEs did not differ among age groups. It must be noted that due to the small number of patients per subgroup, no meaningful conclusions can be drawn from this analysis.

The incidence rates of potentially fatal irAEs were 14, 20, and 9 (per 100 person-years) for patients < 65 years, 65–74 years and ≥ 75 years, respectively (supplementary Table 5). As shown in Fig. 4C, the pooled analysis of all the investigated irAEs per subgroup showed no statistically significant difference between age and the incidence of these irAEs.

The rates of systemic corticosteroid requirement (to treat irAEs) were 143, 123 and 100 (per 100 person-years) for patients < 65 years, 65–74 years and ≥ 75 years, respectively (supplementary Table 5). This indirect method of estimating the incidence of irAEs among the patients showed a decreased need for systemic corticosteroid treatment with increased age, which was statistically significant among the oldest group of patients (RR 0.70, 95% CI 0.56–0.87) (Fig. 4D).

## Discussion

This real-world retrospective study investigated the effectiveness and safety of PD-(L)1 inhibitors in older patients with stage III and IV NSCLC using text mining in the EHR. To the best of our knowledge, this is one of the few studies that has utilized text-mining to collect real-world data, in real-time, of such a large cohort of patients. Median age at the start of immunotherapy was 66 years, which was higher than in pivotal studies [4, 5, 8]. Although, the median PFS and median OS of patients aged 65–74 were four and five months shorter than the youngest patient group respectively, no statistically significant association was observed between age and the effectiveness of anti-PD-(L)1 therapy. The median PFS and median OS were highly comparable between the youngest and the oldest patients. Similarly, older age did not negatively impact the safety of PD-(L)1 inhibitors. In fact, the cumulative incidence of potentially fatal irAEs and the relative risk of corticosteroid prescription to treat irAEs seemed to be lower in patients aged 75 and older.

Similar to our findings, subgroup analyses of pivotal trials and real-world studies have shown no association between age and clinical outcomes (i.e. PFS and OS) [23–27]. However, most large studies have defined “older patients” as those aged ≥ 65 years or ≥ 70 years[28, 29], reducing the certainty of the findings in patients older than 75 years [26, 30]. A pooled analysis of phase III trials for pembrolizumab demonstrated that the median OS was comparable between patients older than 75 years and their younger counterparts, which is in line with the findings of this study [16]. However, other studies have shown either longer [31] or shorter [30, 32] PFS among patients ≥ 75 receiving PD-(L)1 inhibitors as a treatment for NSCLC. Most of these studies are performed in small cohorts and need to be further validated in larger cohorts. The proportion of patients with a ECOG PS ≥ 2 is generally lower in our study compared to the abovementioned studies; this trend is similar among all the different age groups.

The association between age and effectiveness outcomes remained statistically insignificant even when age was analyzed as a linear variable. Additionally, a higher proportion of elderly patients received PD-(L)1 inhibitors as first line treatment in our cohort. This group also had a higher PD-L1 expression. Although both of these factors could potentially improve response rates, subgroup analysis by treatment line and PD-(L)1 expression showed no significant difference in effectiveness outcomes for the older patients compared to younger patients.

With regards to safety of anti-PD-(L)1 antibodies, the most commonly occurring potentially fatal irAE in our study was pneumonitis (5.2%), which lies within the range of the incidence of any-grade pneumonitis in pivotal RCTs (1–10%) [4, 5, 8, 33]. The incidence of pneumonitis was highest among patients aged 65–74 years in this cohort. As for the other studied irAEs, we observed a higher incidence compared to the reported incidences in clinical trials [4, 5, 8, 34, 35]. This was to be expected as RCTs are generally not adequately powered to detect rare events. Our findings were consistent with the reported incidences in other real-world studies [35–37].

Similar to the results of a number of retrospective studies, we found no difference in the incidence of potentially fatal irAEs among different age groups. There are studies suggesting a higher incidence of grade ≥ 2 [24] and ≥ 3 [37] irAEs in patients aged ≥ 70 years. However, the findings of a meta-analysis performed by the Food and Drug Administration (FDA) [38] on the safety of nivolumab in older patients suggested that certain irAEs (i.e. pneumonitis and hepatitis) are more common among younger patients (< 65 years), while other irAEs (i.e. colitis and nephritis) occur more frequently in older patients (≥ 65 years). In our study a decrease in systemic corticosteroid use was observed during immunotherapy in patients aged 75 and older, indirectly suggesting a possible lower incidence of moderate-severe irAEs in older patients. Alternatively, this finding could be the result of the reluctance of the physicians in prescribing corticosteroids for older patients owing to their unfavorable safety profile. This finding must therefore be interpreted with caution.

Generally speaking, older adults have a lower life expectancy. In other words, mortality rate among older patients must be inherently higher than in younger patients irrespective of the intervention that is being investigated. Although the magnitude of this effect cannot be determined in patients with advanced stages of cancer, one could argue that a similar survival regardless of age suggests a higher effectiveness of PD-(L)1 inhibitors in older patients. On the other hand, one possible explanation for the improved safety of anti PD-(L)1 antibodies, in terms of reduced corticosteroid use, in older patients is the reduced sensitivity of the immune system with increased age. However, this theory could only be true if PD-(L)1 inhibitors also showed a lower effectiveness in older patients, which contradicts our findings as well as the abovementioned theory. In addition, preclinical studies suggest that immunosenescence results in higher concentrations of inflammatory cytokines and autoantibodies which may paradoxically increase the risk of irAEs in the elderly.

The contradictions in our findings can best be interpreted in two ways. First, the disease itself is the primary determinant of life expectancy in stage III/IV NSCLC due to the low survival rates, thus making the pure effect of age negligible. Second, oncologists may be more cautious when prescribing immunotherapy to older patients as they may have concerns about comorbidities, reduced tolerance and potential immunotherapy-related toxicities, introducing an inherent selection bias in patient population. Therefore, it may seem plausible to think that the older patients in this study would be relatively fitter (with a longer life expectancy) than the general elderly population with stage III/IV NSCLC. Be that as it may, more older patients had a poor performance score (≥ 2) at the beginning of therapy compared to younger counterparts. We do appreciate that ECOG PS is not the only indicator of frailty and other, unmeasured, factors also play a role in determining it.

This study has several limitations. First, we included a heterogeneous patient population receiving different types of PD-(L)1 inhibitors in various treatment regiments. The effectiveness and toxicity profiles of ICIs may depend on the drug type and the concurrent use of other anti-cancer therapies. Due to the limited number of patients in each treatment subgroup it was not possible to perform stratified analysis with a high enough power. Consequently, a direct comparison between our findings and the outcomes of RCTs was also not possible. Nevertheless, our results are generally comparable to those previously reported in the literature and add valuable information on the effectiveness and toxicity of ICIs in older patients with NSCLC. Second, we used the need for corticosteroid treatment as an indirect measure of the toxicity profile of PD-(L)1 inhibitors. Although the majority of moderate/serious irAEs are commonly treated with corticosteroids, this is not always the case. In addition, patients receiving the corticosteroid dexamethasone for an antiemetic indication were not excluded from the analysis. This is only relevant for patients receiving chemo-immunotherapy with a moderate-high emetic risk regimen (e.g. cisplatin and carboplatin) as dexamethasone is not indicated for low emetic risk treatments such as PD-(L)1 inhibitors. The proportion of patients receiving chemo-immunotherapy is comparable between the three age groups. Furthermore, the incidence of TNF-alpha inhibitor use to treat irAEs could not be determined dur to the insignificant number of patients.

Other limitations include the retrospective nature, the limited data availability on factors such as the genetic markers and comorbidities, and the considerable rate of missing data for ECOG PS and BMI. In order to handle the missing data on the aforementioned factors, multiple imputation was used during analysis. It seems plausible to assume that less severe or absent outcomes are more likely to be overlooked and be left unrecorded in the EHR. The multiple imputation of the missing data could therefore potentially lead to bias and overestimate the number of cases with less favorable patient parameters.

In this study we investigated the impact of age on the effectiveness and irAEs of PD-(L)1 inhibitors in patients with NSCLC. Aging is often associated with frailty. However, it goes without saying that although frailty is common in older adults, it is certainly not always a manifestation of old age. Above chronological age, frailty depends on factors like comorbidity burden, physical status, unhealthy life style and polypharmacy. Studying frailty may therefore provide more valuable data. However, much of the data on frailty was either absent in patient files or not feasible to extract using CTcue.

In spite of these limitations, this study provides important “real-world” information about the impact of age on the effectiveness and the safety of ICIs in a very large cohort of patients with stage III and IV NSCLC. Additionally, the real-world data for this study was obtained using a novel text-mining method.

## Conclusion

In this large cohort real-world study, we used a novel text-mining technique to investigate the effect of age on the long-term effectiveness and irAEs of PD-(L)1 inhibitors in patients with stage III and IV NSCLC. All patients, regardless of age, had similar effectiveness outcomes and comparable risk of moderate-severe and potentially fatal irAEs. These findings suggest that anti-PD-(L)1 antibodies could effectively and safely be prescribed to older patients with stage III/IV NSCLC.

The successful utilization of a text-mining tool to obtain all the data for this real-world study demonstrates the capacity of this efficient technique to extract valuable pieces of information from structured and unstructured fields of the EHR.

Future studies should be performed in larger patient populations in order to be able to investigate the effect of individual immunotherapy agents and treatment regimens with high power. Additionally, a better way to characterise “older” adults in such studies may be to investigate the degree of frailty (based on geriatric assessments) instead of chronological age, as frailty may be a limiting factor for prognosis of patients receiving immunotherapy.

## Supplementary Information

**Additional file 1.**

## Data Availability

All data generated or analysed during this study are included in this published article and its supplementary information files.

## Abbreviations

irAE: Immune-related adverse events
PD-(L)1: Programmed death-(ligand)1
NSCLC: Non-small cell lung cancer
PFS: Progression-free survival
OS: Overall surviva
HR: Hazard ratio
ICI: Immune checkpoint inhibitor
RCT: Randomised clinical trials
WHO: World Health Organisation
SmPC: Summary of product characteristic
RWE: Real-world evidence
EHR: Electronic health records
NLP: Natural language processing
CDC: Clinical data collector
METC: Medical Ethics Review Committee
DTC: Diagnosis treatment combinatio
CMS: Cytostatic Management System
CI: Confidence interval
RR: Relative risks
FDA: Food and Drug Administration

## Footnotes

1 A DTC is a code used for hospital costs reimbursement in The Netherlands.
